# Specific Relationship between the Shape of the Readiness Potential, Subjective Decision Time, and Waiting Time Predicted by an Accumulator Model with Temporally Autocorrelated Input Noise

**DOI:** 10.1523/ENEURO.0302-17.2018

**Published:** 2018-02-12

**Authors:** Aaron Schurger

**Affiliations:** 1 Cognitive Neuroimaging Unit, INSERM, Gif sur Yvette 91191, France; 2 Commissariat à l’Energie Atomique, NeuroSpin Center, Gif sur Yvette 91191, France

**Keywords:** autocorrelation, Bereitschaftspotential, leaky stochastic accumulator, readiness potential, voluntary action

## Abstract

Self-initiated movements are reliably preceded by a gradual buildup of neuronal activity known as the readiness potential (RP). Recent evidence suggests that the RP may reflect subthreshold stochastic fluctuations in neural activity that can be modeled as a process of accumulation to bound. One element of accumulator models that has been largely overlooked in the literature is the stochastic term, which is traditionally modeled as Gaussian white noise. While there may be practical reasons for this choice, we have long known that noise in neural systems is not white – it is long-term correlated with spectral density of the form 1/*f*^β^(with roughly 1 < β < 3) across a broad range of spatial scales. I explored the behavior of a leaky stochastic accumulator when the noise over which it accumulates is temporally autocorrelated. I also allowed for the possibility that the RP, as measured at the scalp, might reflect the input to the accumulator (i.e., its stochastic noise component) rather than its output. These two premises led to two novel predictions that I empirically confirmed on behavioral and electroencephalography data from human subjects performing a self-initiated movement task. In addition to generating these two predictions, the model also suggested biologically plausible levels of autocorrelation, consistent with the degree of autocorrelation in our empirical data and in prior reports. These results expose new perspectives for accumulator models by suggesting that the spectral properties of the stochastic input should be allowed to vary, consistent with the nature of biological neural noise.

## Significance Statement

The cortical readiness potential (RP) is a gradual buildup of scalp electrical potential, and underlying neural activity in motor areas, that reliably precedes the onset of voluntary self-initiated movements by up to one second or more. More than fifty years after its discovery, the functional nature of the RP remains unclear. Here I argue, based on empirical evidence, that the RP reflects the stochastic input to an accumulation-to-bound decision process, and that this stochastic input is temporally autocorrelated, and not Gaussian white noise as it is traditionally modeled. The argument is supported by testing and confirming two novel predictions that emerge from an accumulator model when the stochastic input noise is autocorrelated rather than white.

## Introduction

Uncued voluntary movements are preceded reliably by a slow buildup of cortical activity known as the Bereitschaftspotential or readiness potential (RP; Shibasaki and Hallett, 2006). Since its discovery in the 1960s (Kornhuber and Deecke, 1965), the RP has been interpreted as a sign of movement preparation, the outcome of a preconscious neural decision to initiate an action (Libet et al., 1983). Recent evidence, however, supports a different interpretation: the RP reflects ongoing stochastic fluctuations in neural activity that favor the spontaneous emission of a movement at certain times more so than at others (Schurger et al., 2012; Murakami et al., 2014; Schmidt et al., 2016). Both the shape of the RP and the distribution of waiting times (how long the subject waits before producing a spontaneous movement) can be well described by a leaky stochastic accumulator model (Schurger et al., 2012; Murakami et al., 2014).

**Figure 1.**
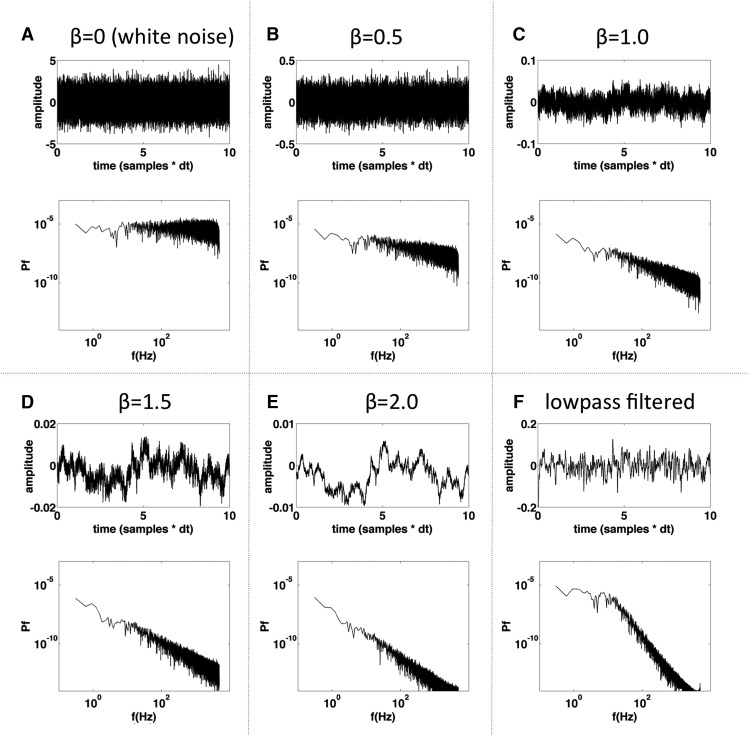
Illustration of white noise and different flavors of pink noise. ***A–E***, Noise with different 1/*f* exponents (β, where spectral power scales as 1*f*^β^ in log-log coordinates). The upper axis of each panel shows the time series and the lower axis shows the power spectrum; β specifies the slope of the power spectrum in units of log power versus log frequency, so if β = 0, then the noise is white (flat power spectrum, roughly equal power in all frequencies, each sample independent of all other samples). Notice that as β increases, the time series becomes more and more dominated by low-frequency fluctuations, as indicated by the slope of the spectra. Time series are shown for values of β between 0 and 2 to illustrate the way that time series change qualitatively as β changes. ***F***, Same noise series as in **A** after lowpass filtering with a first-order Butterworth filter (cutoff frequency of 1 Hz).

Accumulator models at a minimum consist of a constant term (reflecting the decision evidence) plus a stochastic term (see Materials and Methods). Integration over these “inputs” to the accumulator results in the “output” of the accumulator, commonly referred to as the decision variable because a decision is made and action initiated when the output exceeds a certain threshold. One element of accumulator models that has been largely overlooked in the literature is the stochastic term, which is traditionally modeled as Gaussian white noise. While there may be practical reasons for this assumption, it is well known that noise in neural systems is not white, but instead is pink – it is long-term correlated with spectral density of the form 1/*f*^β^(with roughly 1 < β < 3; β= 0 for white noise) across a broad range of spatial scales (Fig. 1; Pereda et al., 1998; He et al., 2010). Whether or not accumulator models should account for the spectral properties of neural noise is an open question. At the same time, we can also ask whether or not the stochastic term in such models can be mapped onto a well-defined neural phenomenon.

A recent study found evidence for an accumulator process in area M2 of rats (homolog of human premotor cortex) performing a task where the rat could spontaneously abandon waiting for a large reward and instead opt for an immediate and certain, but smaller reward (Murakami et al., 2014). Using single-unit recordings, these authors were able to identify two different and interspersed functional subtypes of neurons: ones that behaved like the inputs to an accumulator, with tonic firing rates proportional to the length of time that the rat waited, and others that behaved like the outputs of an accumulator, with firing rates ramping up to a fixed threshold level just when the rat withdrew from the waiting station and sought out the small but certain reward. Which of these two is more likely to dominate in signals picked up by a scalp electrode, the vantage point from which the RP is commonly measured, is unknown.

Thus, here I take up two distinct but related points: (1) that accumulator models of decision-making should allow the slope (β) of the noise spectrum to vary instead of using white noise (Fig. 1), especially when the imperative (drift term) is weak relative to the noise; and (2) that a buildup in event-preceding EEG potential might reflect the average stochastic input to an accumulator process (“RP-as-input”) rather than its average output (“RP-as-output”).

With the above two points in mind, I explored the behavior of a leaky stochastic accumulator when the noise over which it accumulated was temporally autocorrelated, and empirically tested two novel predictions that emerged. I tested these two predictions on behavioral and EEG data recorded while subjects performed Libet’s self-initiated movement task (Libet et al., 1983). One prediction was that the shape of the RP should vary in a specific way as a function of the waiting time (how long the subject waits before producing a spontaneous movement). The second prediction concerned the subjective estimate of the time of the “urge” to move, what Libet et al. (1983) referred to as ‘W’ time: W time was predicted to vary as a function of waiting time, becoming earlier (with respect to movement onset) with longer waiting times. Both predictions were confirmed, with the model suggesting biologically plausible levels of autocorrelation (*β* ≅ 1.4). Taken together, the results suggest that the EEG RP may reflect the autocorrelated stochastic input to an accumulation-to-bound process, and highlight the potential utility of using biologically realistic noise in accumulation-to-bound models.

## Materials and Methods

I reanalyzed behavioral and EEG data from a prior study (Schurger et al., 2012). Details about human subjects, stimuli and task, and data preprocessing are summarized below. Details about data analyses, statistics, and computational modeling are reported in full.

### Human subjects

A total of 16 subjects participated in the study (six female, mean age 28, one left handed). Subjects were paid for their participation and all gave written informed consent. Two of the subjects did not exhibit a RP (a negative trend in electrical potential preceding movement onset at Cz or any adjacent electrode) and so were excluded from further analyses, leaving *N* = 14.

### Stimuli and task

Subjects sat ∼60 cm in front of a translucent screen onto which the stimulus was back-projected. The subject sat in a reclined position with dim ambient lighting. The stimulus was a small clock face (white on a black background) with a diameter of ∼ 6° of viewing angle. A small white dot circled smoothly around the edge of the clock dial, completing one full cycle every 3 s (equivalent to 50 ms per tick mark on the clock). Each trial began with the onset of the clock rotation. Subjects were instructed to wait for the clock to complete one full cycle. After that the subject was free to perform the instructed movement (pressing a button with the thumb of the dominant hand) at any time. Subjects were encouraged to try not to preplan the moment when they would press the button, but rather to do so spontaneously without any forethought (Libet et al., 1983). After pressing the button, the dot continued to circle the clock face for one more second and then the screen went blank. Subjects were instructed that after the screen went blank they should report (verbally) what had been the position of the dot at the moment that they first became aware of their urge to press the button. In addition to this task (the “classic” task) subjects also performed a second task which was identical except that on some trials subjects were interrupted by an auditory “click” indicating that they should press the button immediately (the “interruptus” task). The analyses reported here were performed on a combination of the data form the first task and the uninterrupted trials from the second task.

### EEG data acquisition

EEG data were recorded inside of a shielded room using a 60-channel EEG system (Elekta NeuroMag EEG/MEG system) sampled at 1000 Hz. Of the 60 channels, I used a subset encompassing the standard 10-20 montage, with the addition of electrodes C1, C2, FC1, and FC2.

### EEG data preprocessing

Data preprocessing and analysis were performed using Matlab (MathWorks Inc) along with the FieldTrip toolbox for Matlab (Oostenveld et al., 2011). Data were first downsampled to 250 Hz. Independent component analysis (ICA) was used to identify and remove ocular artifacts from the data (Jung et al., 2000), and trials with artifacts remaining after this step were excluded by visual inspection. I extracted data epochs time locked to the first button press after the start of the trial. Each data epoch covered the window of time extending from -3.5 to +1.0 s relative to the button press.

### Data analyses and statistics

#### Behavioral

I define the “waiting time” (or just “wait time”) as the amount of time, in seconds, that elapsed from the beginning of the trial until the subject first pressed the button. ‘W’ time (Libet et al., 1983) was the time, relative to the onset of the button press, that the subject reported first having been aware of the urge or decision to press the button. This was recorded by taking the clock time at which the subject reported having had the urge and subtracting it from the clock time of the actual movement. The correlation between waiting time and W time was computed using Pearson’s correlation coefficient applied both to the pooled data from all subjects and also separately for each subject. In the latter case, the resulting *r* values across subjects were tested for significance (difference from zero) using Wilcoxon's signed rank test.

### EEG

EEG data epochs were sorted according to waiting time into the lower 33rd percentile (short waiting time) and the upper 33rd percentile (long waiting time), and averaged together within each group to compare the shape of the RP for short and long waiting times. Differences in the amplitude of the RP at each time point were tested for significance using a signed rank test and then subjected to a cluster-based permutation test to correct for multiple comparisons. Unless specified otherwise, the RP was measured at electrode C1 (C2 if left handed).

#### Leaky stochastic accumulator model

Accumulator models at a minimum involve integration over an input signal (drift or “imperative”) plus Gaussian white noise. Noise in the brain, however, is temporally autocorrelated with spectral density of the form 1/*f*^β^ with typically 1 < β < 3 (“pink” noise). The model that I used was the same as that used in prior studies (Schurger et al., 2012; Murakami et al., 2014), namely the leaky stochastic accumulator model (Usher and McClelland, 2001), except that the noise was pink, with *β* allowed to vary arbitrarily, instead of white (*β* fixed at 0). Performing a run of the simulation amounts to performing numerical integration over the following differential equation:

$$dx=\left(I-kx\right)dt+c\xi_{\beta }\sqrt{dt}$$

where *I* is the drift rate (the imperative to move), *k* is leak, and ξ_β_ is noise with 1/f exponent β allowed to vary arbitrarily, from 0 (white noise) to 3; *c* is a noise scaling factor (by convention *c* = 0.1). I sometimes refer to the output of the accumulator (*x* in the equation above) as the decision variable. After each run of the simulation I extracted two “data epochs,” one from the noise input to the accumulator and one from the output of the accumulator. Both epochs were time aligned to the sample at which the output of the accumulator first crossed the threshold, and spanned the interval from 5000 samples before the crossing to 500 samples after. When I fit the event-locked input to the accumulator to the shape of the RP, I use the term RP-as-input, and when I fit the event-locked output of the accumulator to the shape of the RP, I use the term RP-as-output. The simulation is one and the same in both cases, the only difference being which by-product of the simulation (the input or the output) is considered to represent the RP.

#### Generation of pink noise

Normally, when simulating an accumulator process, a series of computer-generated Gaussian-distributed pseudo-random numbers is used to instantiate the (white) noise term. For optimal performance it is best to generate the entire time series of pseudo-random numbers all at once at the beginning of the simulation, and then step through it, rather than generating a single random number on each iteration. Having the entire time course of the noise in hand allows one to change the spectral properties of the noise as desired. Power-law or 1/*f* noise has spectral power that is inversely proportional to frequency, roughly following a negative-sloping line in log-log coordinates (He et al., 2010; Fig. 1): *P* ∝ 1/*f*^β^ with *β* commonly referred to as the “1/*f* exponent.” For the simulations reported here, I first generated a time series of Gaussian pseudo-random numbers and then altered the spectrum of this noise as follows: first the signal was converted into the frequency domain via a fast Fourier transform (*fft* in Matlab); then a given (negative) slope was imposed on the power spectrum by multiplication with a log-linear function having the desired slope; and then the signal was converted back into the time domain via an inverse Fourier transform (*ifft* in Matlab). The resulting time series, which could have any arbitrary *β* (for examples, see Fig. 1), was then used as the noise input to the simulated accumulator process. Note that I make no assumptions or claims about the spectral properties of the time series other than that they must be temporally autocorrelated. The procedure described above is simply a convenient way to obtain autocorrelated noise while parametrically varying the degree of temporal autocorrelation in the time series.

#### Data fitting

For separate runs of the simulation, the average time course of the model output or the stochastic input to the model was fit to the average RP across subjects. The time courses were time locked to the first crossing time, which is of course always determined based on the output of the accumulator. For both the RP-as-input and RP-as-output simulations the fitting procedure included a scaling factor whereby the amplitude of the simulated RP was scaled to that of the empirical RP. In each case, the normalized distribution of first-crossing times of the model was simultaneously fit to the normalized empirical waiting time distribution. Fitting was performed using multidimensional unconstrained nonlinear minimization (Nelder-Mead; function fminsearch in Matlab) on the mean-squared error between the simulated and empirical data. Appropriate starting values for the parameters, including scaling parameters, were identified by first trying a few fits by hand.

#### Modeling of ‘W’ time and derivation of the prediction

**Figure 2.**
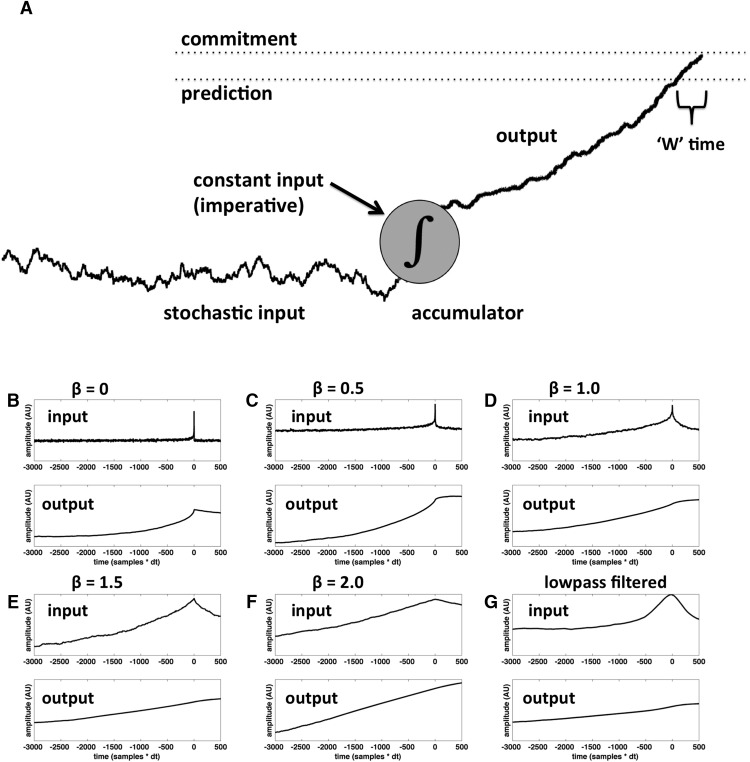
Schematic illustration of the model (***A***) with sample input and output (***B–G***). In ***A***, the ∫ symbol represents the neural accumulator, with pink noise plus constant input on the left and output on the right. The higher of the two thresholds is the activation threshold: when this threshold is crossed, then the “neural decision to move” has been made and movement ensues. The lower of the two thresholds is a self-monitoring threshold: when this threshold is crossed a signal is generated indicating that movement is about to ensue with very high probability. When subjects are asked to estimate the time at which they were first aware of an urge to move (‘W’ time), this decision is (according to the model) informed by the delay between the crossing of the two thresholds. ***B–F***, Average input and output from the model for different values of the 1/f exponent (β). ***G***, Same for lowpass filtered white noise (see Materials and Methods).

I modeled the subjective estimate of the time of the conscious urge to move (Libet’s ‘W’ time) by adding a second threshold, slightly lower than the main activation threshold (Fig. 2), similar to Ganos et al. (2015). This “advance warning” threshold is sufficiently close to the main threshold that crossing it is a good predictor that the main threshold is about to be crossed (i.e., movement is about to ensue) with very high probability. ‘W’ time in this model is simply the temporal delay between the crossing of the two thresholds, expressed with respect to the crossing of the second threshold (i.e., a negative number). Note that I do not assume that this predictive information uniquely determines ‘W’ time, but only that it informs ‘W’ time. Prior evidence indicates that neural information from both before and after movement onset can influence ‘W’ time (Sirigu et al., 2004; Lau et al., 2007; Banks and Isham, 2009; Douglas et al., 2015), and thus that the brain likely makes use of information from both before and after movement onset in its estimation of ‘W’ time. Here, I only assume that a premovement prediction about an upcoming movement and its reafferent consequences (Wolpert, 1997) is at least part of the information that contributes to the brain’s estimate of ‘W’ time.

#### *Estimation of the* 1/*fexponent*(β)

The 1/*f* exponent was computed as the sign-reversed slope of the best fitting line fit to the fractal component of the power spectrum in log-log coordinates. The fractal component of the power spectrum was estimated using the IRASA method of Wen and Liu (2016) and the best fitting line was estimated using ordinary least squares regression (lscov in Matlab).

#### Code accessibility

Computer code (Matlab) for running the simulations is available as Extended Data 1, and is also available online at https://bitbucket.org/aschurger/lsa_rp_model.

10.1523/ENEURO.0302-17.2018.ed1 Extended Data 1 Download Extended Data, ZIP file.

## Results

### Behavioral

#### Waiting time

Subjects waited on average 7.1 (±0.63 s SEM) seconds to produce a movement in the classic Libet task, and 5.4 s (±0.31 s SEM) in the interruptus task. This difference was significant (*p* < 0.001; two-tailed *t* test, *t* = 4.5, df = 14), although the two distributions were mostly overlapping (see Fig. 3*B* of Schurger et al., 2012). Since I combined noninterrupted trials from the interruptus task with data from the classic task, I performed an additional test (see below) to make sure that this average difference in waiting time did not account for any of the other results related to waiting time.

**Figure 3.**
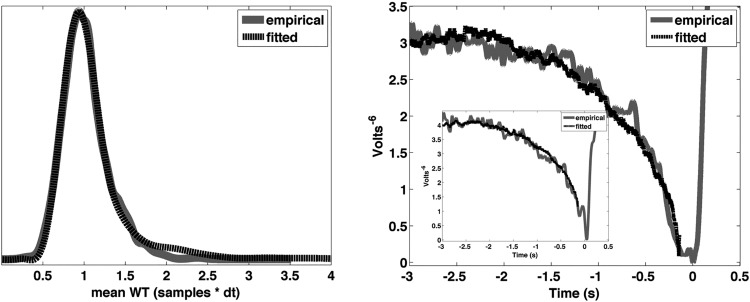
Fitting the model to the data under the RP-as-input interpretation. Left, The distribution of first crossing times (dashed black line) fit to the empirical distribution of waiting times (solid gray line). Right, The (sign reversed) average stochastic input to the accumulator time locked to threshold crossings in the output (dashed black line) fit to the empirical RP (RP at C1; solid gray line). The two fits were performed simultaneously, i.e., with the same parameters. The parameters used for the best fit were β = 1.4, I = 0.1, k = 0.6, and threshold = 0.1256. Inset shows the same fit (same parameters), but with the RP measured at either electrode FC1, C1, or Cz chosen individually for each subject depending on which electrode had the highest amplitude signal at *t*_(0)_. Note that the fitting procedure included a scaling factor whereby the amplitude of the simulated RP was scaled to that of the empirical RP.

#### ‘W’ time

Libet et al. (1983) used the term ‘W’ time to refer to the subjective estimate of the time of the conscious urge to move. The mean ‘W’ time in the classic task was –142 ms (±34 ms SEM), and the mean ‘W’ time in the interruptus task was –126 ms (±44 ms SEM). The ‘W’ times in the two tasks were not significantly different across subjects (*p* = 0.45; two-sided paired samples signed rank test).

### EEG RP

Fourteen of 16 subjects exhibited the gradual negative deflection in scalp electrical potential before movement onset, at or near the vertex, that is characteristic of the RP. The two subjects that did not exhibit an RP were excluded from further analyses. The average RP across subjects (*N* = 14) is shown in Figure 3*B*. The tail of the RP extended back in time at least two full seconds before nearing its horizontal asymptote.

### Data fitting

The RP-as-output variant of the model has been shown previously to fit the data well (Schurger et al., 2012), but what about the RP-as-input variant? Although the present argument is focused on empirical predictions made by the two models, nevertheless it was important to confirm that the RP-as-input model was capable of fitting the data, before going on to test empirical predictions derived from it.

**Figure 4.**
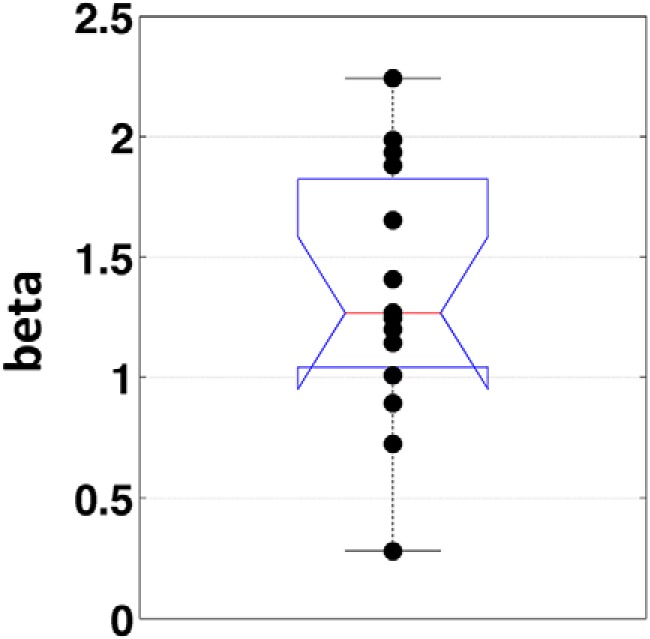
Estimated 1/*f* exponent. Boxplot of the 1/*f* exponent (β) estimated for each subject (*N* = 14) at electrode C1. Each dot represents one subject. See Materials and Methods for the estimation procedure.

The RP-as-input model is in fact capable of producing a very good fit to the data. Figure 3 shows the normalized distribution of first crossing times of the output of the accumulator fit to the normalized empirical waiting time distribution (*n* = 14). Also shown is the average time course of the stochastic input to the accumulator (time locked to threshold crossings in the output), fit to the average RP. The parameters for the best fit were obtained after an exhaustive search of the parameter space, and were as follows: β = 1.4, I = 0.1, k = 0.6, and threshold = 0.1256. Thus, a single set of parameters exists such that the RP is well fit by the event-locked stochastic input to the accumulator and the waiting time distribution is well fit by the first-crossing time distribution of the output. It is noteworthy that the value for β was 1.4, which is approximately the value for β found in our EEG data (1.34 ± 0.14 SEM; Fig. 4) and that has been observed in the past for EEG data (Pereda et al., 1998; reported β = 1.5).

One might ask whether or not it is necessary to use 1/*f* noise as input to the accumulator. Perhaps all that is necessary is to suppress the higher frequencies, in which case a low-pass filter applied to white noise might suffice (Fig. 1*F*). One problem with this reasoning is that this procedure could be seen as simply a very crude way of approximating 1/*f* noise, which would bring us back to the original question. Nevertheless, it is unclear a-priori how well the model would fit the data using low-pass filtered white noise instead of 1/*f* noise as input to the accumulator. I tested this possibility using a first-order Butterworth low-pass filter, varying the cutoff frequency. I used a low-order filter because it has a shallow cutoff, thereby approximating more closely the relationship between frequency and power found in 1/*f* noise (with higher-order filters the fit, in particular to the RP, only became worse). I used the same exhaustive parameter search here as I had used when running the model with 1/*f* noise.

Using low-pass filtered noise as input to the accumulator, under the RP-as-input assumption, the model could fit either the RP or the wait-time distribution, at the expense of the fit to the other, but had trouble fitting both with the same set of parameters. Notably, the best fit overall using low-pass filtered noise was significantly poorer than the best fit achieved with simulated 1/*f* noise (MSE of best fit with low-pass filtered noise 0.0271 ± 0.00066 SD, vs 0.0146 ± 0.00054 SD using 1/*f* noise; *p* < 10^−9^, two-sided Wilcoxon rank sum test). Again, this might simply reflect the fact that a low-pass filter applied to white noise yields a relatively poor approximation of 1/*f* noise. However, as stated previously, I make no claims about the necessity of “true” 1/*f* (power-law) noise, only that the input noise should be temporally autocorrelated, although it would appear that simulated 1/*f* noise does lead to a significantly better fit overall.

### Prediction 1: relationship between the shape of the RP and waiting time

**Figure 5.**
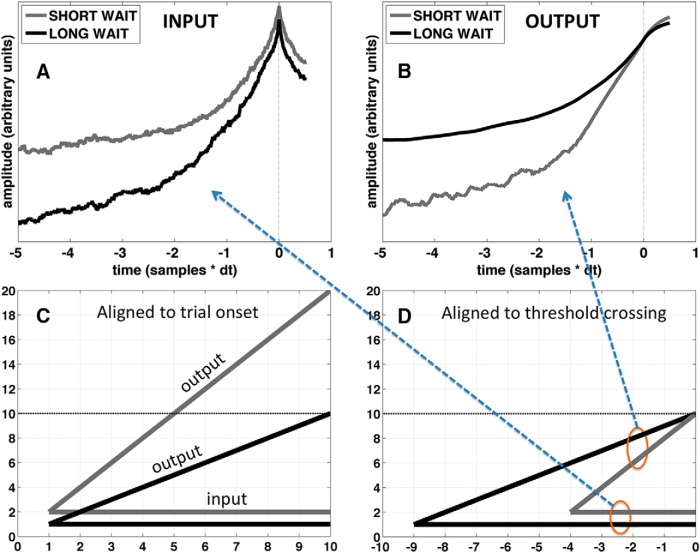
Predicting the shape of the RP as a function of waiting time. ***A***, Average stochastic input to the accumulator, time aligned to first crossing times in the output, separately for trials with a long wait (upper 33rd percentile; black line) and for trials with a short wait (lower 33rd percentile; gray line). ***B***, Same as ***A*** but for the output of the accumulator. The early tail of the output on “short wait” trials is noisier than the rest because of missing data: on trials with a short wait, often the climb to the threshold was shorter than the epoch length. ***C***, Schematic depiction of the input and output for constant input, time aligned to the beginning of the trial. On trials with a short wait, the input is greater and the output rises more quickly to the threshold. ***D***, Same as ***C***, but time aligned to the threshold crossing. Notice that when time is aligned to the threshold crossing the relationship between input and output becomes reversed. This helps to intuitively explain the reversal in the relationship between the predicted shape of the RP for long- and short-wait time trials. Parameters used for ***A***, ***B***: β = 1.4, I = 0.1, k = 0.6, and threshold = 0.1256. However, the relationship between predicted RPs for short versus long wait times (reversal of amplitude relationship for input versus output) remained qualitatively the same regardless of the specific parameters used, as long as β was >∼0.5. Regarding ***A***, ***B***, note that, because the epochs are time locked to threshold crossings in the output, only the outputs (***B***) are guaranteed to reach the same amplitude at *t*_(0)_. The two curves in ***A*** do not necessarily have to reach the same amplitude at *t*_(0)_, because these are the average inputs to the accumulator. The inputs for long and short waits in ***C***, ***D*** are set to 1 and 2, respectively, for illustrative purposes, so that the slope of their respective outputs will be 1 and 2. Note that this overly simplified schematic is only intended to describe the relationship between the input and output but not their shape.

**Figure 6.**
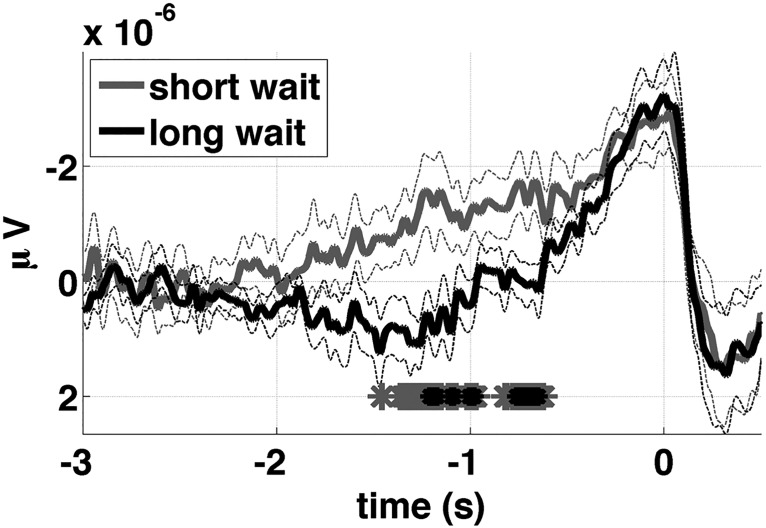
The shape of the RP as a function of waiting time. Average empirical RP (sign reversed for easier comparison with model predictions) at electrode C1 for trials with short (gray line) and long (black line) waiting times. Thin dashed lines show standard error of the mean. Stars at the bottom of the axis mark time points where the difference between the two was significant at *p* < 0.05 (gray stars) and *p* < 0.01 (black stars); *p* < 0.01 for the mean amplitude over the range -1.5 to -0.5 s (two-tailed signed rank test).

The predicted relationship between waiting time and the shape of the RP under the RP-as-input and RP-as-output assumptions is shown in Figure 5, for the parameters reported above (β = 1.4, I = 0.1, k = 0.6, and threshold = 0.1256). Although these specific parameters were the ones that resulted in the best fit under the RP-as-input assumption, the predicted relationship between RPs for short versus long wait times remained qualitatively the same regardless of the specific parameters used, as long as β was more than ∼0.5. As illustrated in the figure, under the RP-as-input assumption the model predicts that the early RP will have a lower amplitude for long versus short waiting times (Fig. 5*A*). On the other hand, under the RP-as-output assumption the model predicts the opposite: a higher amplitude early RP for long versus short waiting times (Fig. 5*B*). The empirical data clearly support the RP-as-input interpretation (Fig. 6; *p* < 0.01 for the mean amplitude over the range -1.5 to -0.5 s, two-tailed signed rank test).

To understand the relationship (Fig. 5*C*,*D*), consider that the output of the accumulator will tend to have a steeper slope on trials with a short waiting time. This translates into a lower initial amplitude, when time aligned to the first crossing time. Whereas trials with a longer waiting time will tend to have a more gradual, shallow slope, and thus a higher amplitude when time aligned to the first crossing time. The input to the accumulator, on the other hand, will tend to have a higher amplitude throughout the epoch for trials with a short waiting time (the higher its amplitude the sooner its integral will cross a given threshold value). This relationship is explained schematically in Figure 5*C*,*D*, which illustrates the relationship for the simplest case where the input is constant and the output is linear. Note that this overly simplified schematic is only intended to describe the relationship between the input and output but not their shape.

### Prediction 2: correlation between waiting time and W time

**Figure 7.**
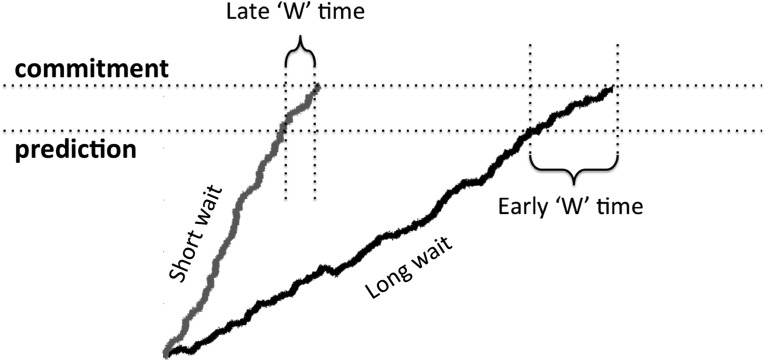
Predicting ‘W’ time as a function of waiting time. Schematic showing how ‘W’ time is predicted to vary as a function of waiting time. On trials with a short waiting time, the slope of the decision variable is steeper (gray line) than on trials with a long waiting time (black line). A steeper slope means that the interval between the crossing of the two thresholds will be shorter, so ‘W’ time will be closer in time to the onset of movement (smaller in absolute value). For trials with a more gradual slope of the decision variable (black line) the reverse is true, ‘W’ time will be further back in time from the onset of movement and larger in absolute value.

**Figure 8.**
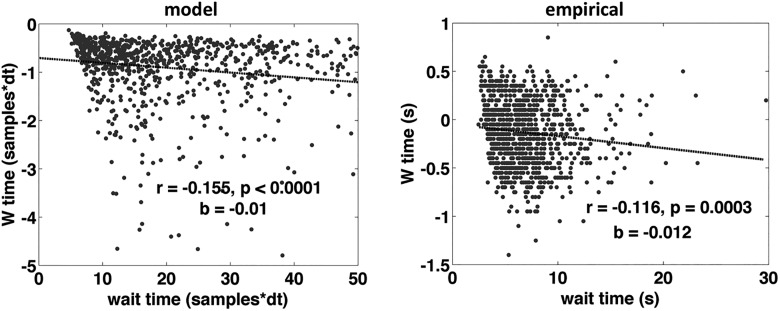
‘W’ time as a function of waiting time aggregate. Correlation between waiting time and ‘W’ time for simulated data from the model (left) and for the empirical data from all subjects (right; each dot is one trial from one subject). Although there is noise in the model, the model data are bounded by zero at the top, because there is no noise in the estimation of ‘W’ time, it is strictly earlier than movement time and is “reported” exactly as is. In reality there is a lot of variance across trials and across subjects in the reporting of ‘W’ time, and this is evident in the panel on the right. Combining the data from all subjects can be problematic, because differences between subjects and differences within subject are confounded, so in Figure 9, I present the correlations separately for each subject.

**Figure 9.**
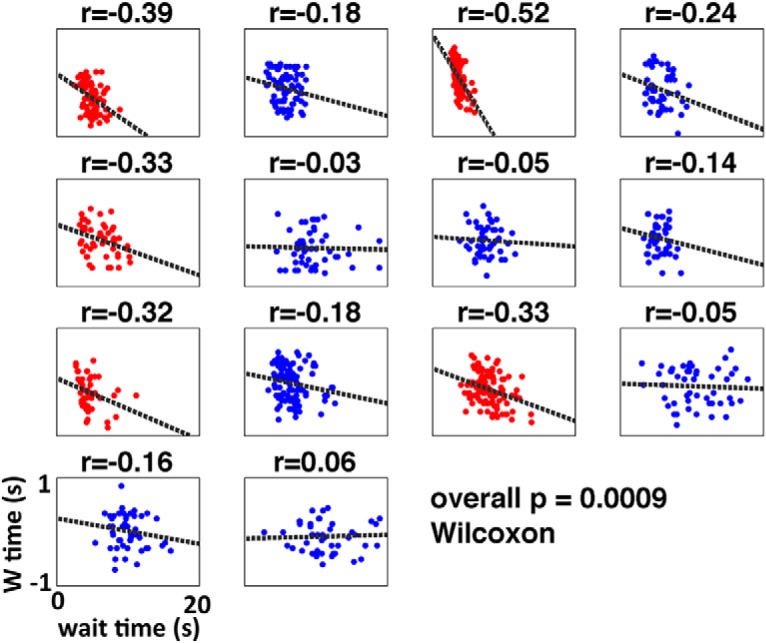
‘W’ time as a function of waiting time per subject. Correlation between waiting time and ‘W’ time for the empirical data grouped by subject. The horizontal axis is the waiting time (from 0 to 20 s) and the vertical axis is ‘W’ time in seconds with respect to movement onset. Data are shown in red if the correlation is individually significant at *p* < 0.05. When the *r* values for all subjects are submitted to a Wilcoxon signed rank test the effect is significant at *p* < 0.001. Also, the probability of 13 subjects (out of 14) individually exhibiting a negative correlation is 0.0009 (binomial test).

As mentioned in Materials and Methods, I assume that the temporal delay between the crossing of the advance warning threshold and the main activation threshold contributes to the subjective estimate of ‘W’ time (Fig. 2). Thus, if the trajectory of the decision variable is relatively steep, then that temporal delay will be relatively short, and so ‘W’ time will be later in time (closer in time to the onset of movement). On the other hand, if the trajectory of the decision variable happens to be relatively gradual, then the temporal delay will be longer and ‘W’ time will be earlier (farther back in time from the onset of movement). Therefore, the prediction is that waiting time should be negatively correlated with ‘W’ time, with longer waiting times predicting earlier ‘W’ times and vice versa (Fig. 7). Remarkably, this prediction is confirmed, both in the pooled data from all subjects (Fig. 8; Pearson’s correlation coefficient: *r* = -0.116, *p* = 0.0003; slope of regression line: b = -0.012), and in the aggregate of individual correlations across subjects (Fig. 9; *p* = 0.0009, two-sided Wilcoxon signed rank test on the *r*’s from each individual subject, *n* = 14; *r* was negative for 13 of 14 subjects, *p* = 0.0009 binomial).

The parameters used to generate the above prediction were the same as those used for fitting the data and predicting the shape of the RP: *β* = 1.4, I = 0.1, k = 0.6, and threshold = 0.1256. The prediction was robust to small changes in the parameters. Most importantly the prediction only held for *β* more than ∼1.2, i.e., this prediction depends on the stochastic input being pink. Thus, allowing β to vary revealed a novel prediction that would not have been revealed otherwise.

The advance warning threshold may be thought of as analogous to the processing of an “efference copy” by an internal forward model (Wolpert, 1997). When the prediction threshold is crossed, information is generated indicating that a specific movement is just about to happen with high probability. After the movement is completed, this information informs the subsequent subjectively-estimated ‘W’ time. Note again that I do not assume that the predictive signal uniquely determines ‘W’ time, but only that it informs ‘W’ time. There is strong evidence that neural information from both before and after movement onset can influence ‘W’ time, and thus that the brain likely makes use of information from both before and after movement onset in making this particular judgment (Lau et al., 2007; Banks and Isham, 2009; Douglas et al., 2015). Here, I assume that a premovement prediction about an upcoming movement (and its reafferent consequences) is at least part of the information that contributes to the brain’s *post hoc* estimate of ‘W’ time.

As mentioned before, to guard against the possibility that this correlation might be driven by the difference in mean waiting time for the classic and interruptus tasks (recall that noninterrupted trials from the interruptus task were combined with data from the classic task), I tested the pooled correlation separately for the data from the classic task and the data from the interruptus task. The correlation was negative and highly significant in both data sets (classic task: *r* = -0.1159, *p* < 0.001; interruptus task: *r* = -0.1156, *p* < 0.001), thus arguing against this possible interpretation.

## Discussion

Here, I explored the behavior of an accumulator model when the power spectrum of the input noise was allowed to vary from white (the standard for such models) to pink, i.e., allowing for varying levels of temporal autocorrelation in the noise. The 1/f exponent (β) of the noise spectrum thus becomes a parameter of the model, which can take values in the range 0 ≤ β < 3. Previous work has looked at stochastic differential equations that can generate autocorrelated noise in their output with a variable 1/*f* exponent (Ruseckas and Kaulakys, 2010), but this is different from the present work which used autocorrelated noise in the input to the process (in place of the Gaussian white noise used by convention).

With the 1/*f* exponent (β) of the input noise as a parameter, I tested the possibility that the canonical RP reflects the average event-locked stochastic input to an accumulator rather than its average output. I first showed that the model, under the RP-as-input assumption, was able to fit the data and then tested a prediction regarding the shape of the RP. Finally, I also tested a prediction regarding ‘W’ time that depends on the input noise being autocorrelated.

Intuitively one might expect that, from the perspective of a scalp electrode, the output of a neural accumulator might be too faint to be detected, because it might be computed by a small population of neurons (compare O'Connell et al., 2012). At the same time, the stochastic input to the decision process should be at least as readily measured at the scalp if it is shared among a large number of neurons spanning a functional network (Mantini et al., 2007). Whatever the case may be, the model provided a way to distinguish between these two possibilities by generating opposing predictions depending on the substrate (input or output) of the observed average (Fig. 5).

The model also incorporated a second “warning” threshold, slightly lower than the primary activation threshold, such that when the lower threshold is crossed the probability of soon crossing the primary activation threshold is high (Fig. 7). This lower threshold allows the model to account for the estimated time of the subjective urge to move (‘W’ time) in terms of the delay between the crossing of the two thresholds. This is clearly an oversimplification of the true state of affairs: prior data have implicated factors from both before and after movement onset in the subjective estimation of ‘W’ time (Sirigu et al., 2004; Lau et al., 2007; Banks and Isham, 2009; Desmurget et al., 2009; Douglas et al., 2015). However, the model is only intended to account for one such factor, originating before movement, plausibly the generation of a forward model (Wolpert, 1997). There has been a great deal of controversy surrounding the meaning of ‘W’ time and its relationship to the onset of intention (for review, see Maoz et al., 2015).

Note that the argument here is not that pink noise is superior to white noise in stochastic accumulator models. Rather it is an argument in favor of allowing β to vary (as it does in real neural systems) in accumulator models of decision-making. The resulting value for β that is suggested by the model could in principle be near zero (i.e., white noise) or it could be roughly between 1 and 2 (pink noise). The point is that having β as a parameter adds a new dimension to the model allowing it to account more fully for the neural data, by fitting its spectral properties. One advantage of doing so is that the stochastic input may then behave in a way that is amenable to modeling, e.g. the the RP-as-input variant of the model can only fit the data when β is non-zero. Another advantage is that with variable β, we can account for properties of the data that were previously overlooked, potentially leading to new predictions.

Allowing the spectral properties of the input noise to vary here led to two novel predictions. The first prediction was that the shape of the RP should vary as a function of the time elapsed between the beginning of the trial and the onset of the self-initiated movement (the waiting time). The relationship between the shape of the RP and waiting time was reversed depending on whether the RP was modeled as the average input to or average output from the accumulator (Fig. 5) thus constituting a strong test with which to adjudicate between these two possibilities. The second prediction was that the estimated time of the subjective urge to move, with respect to movement onset, should be negatively correlated with the waiting time, being earlier in time (with respect to movement onset) for longer waiting times, and vice versa. Both of these predictions were confirmed, lending support to the hypotheses encapsulated in the model. The results are important because they drive a wedge between two different possible (and plausible) interpretations of the RP: (1) as the average event-locked stochastic input to an integration-to-bound process, or (2) as the average event-locked output from an integration-to-bound process. Both results point to the former interpretation.

Note that Caspar and Cleeremans (2015) found that the amplitude of the RP was lower among subjects with late ‘W’ times (“short W group”) compared to subjects with early ‘W’ times (“long W group”), whereas Haggard and Eimer (1999) found the opposite. These studies did not group their data according to waiting time as I have done here, but one can infer the following: If longer waiting times are associated with both earlier ‘W’ times and a lower-amplitude early RP, then early ‘W’ times may be associated with a lower-amplitude early RP. This is broadly consistent with Caspar and Cleeremans (2015), although the difference here was in the early RP, whereas these two prior studies report a difference in the amplitude of the RP overall. Note also that the results of both Haggard and Eimer (1999) and Caspar and Cleeremans (2015) depend on the choice of baseline which has recently been a topic of debate (Khalighinejad et al., 2018). I did not apply any baseline correction to the EEG data reported here.

The hypotheses tested in the present study are based on two premises. One is that, when movements are made spontaneously and voluntarily, a subset of neurons in premotor areas behave like inputs to an accumulator while other neurons behave like outputs (Murakami et al., 2014). This first premise raises the question of which subpopulation of neurons is responsible for the RP, as captured from the perspective of a scalp electrode, or other mass-action recording modality. The second premise is that real biological noise in the brain is not white, but rather has (approximately) a 1/*f* spectral profile. Although we have known about the RP for a long time, attempts to model it mechanistically have come about only recently (Schurger et al., 2012). These two premises expand the horizon of stochastic accumulator models, especially in the low-SNR regime, and add a new dimension to the kinds of hypotheses that can be derived from such models.

The model developed here accounts for both the shape of the RP and W time in terms of a third variable, the time spent waiting to produce a movement. To date, this variable has been given very little attention in the literature, but was predicted to be a factor by the model. To the extent that the waiting time in a self-initiated movement task can be considered analogous to the reaction time in a perceptual decision-making task, we can then consider some of the parallels between them. From the point of view of the accumulator model, both reaction time and waiting time reflect the very same thing: the time it takes for the integral of evidence plus noise to reach a decision boundary. Both produce a gamma distribution, and both exhibit a positive linear relationship between the standard deviation and the mean (Schurger et al., 2012).

The simplifying assumption of Gaussian white noise in accumulator models has held strong for decades, and such models have proven themselves very capable of accounting for behavioral and neural data (Ratcliff, 1978; Ratcliff et al., 1999; Gold and Shadlen, 2001; Usher and McClelland, 2001; Ratcliff et al., 2004; Gold and Shadlen, 2007; Ratcliff and Starns, 2009, 2013). This begs the question of why we should further complicate things by introducing varying levels of autocorrelation in the input noise. The answer is simple: stochastic fluctuations in neural systems tend to be temporally autocorrelated (Destexhe et al., 2003; Rudolph and Destexhe, 2003; Henrie and Shapley, 2005; Garcia-Perez et al., 2007; Milstein et al., 2009; Van De Ville et al., 2010; He, 2011). A real neural integrator, given that it must integrate over fluctuations originating elsewhere in the brain, will likely integrate over autocorrelated fluctuations, and if so then the level of autocorrelation will likely vary between different cognitive states and between different individuals (He et al., 2010). Thus, the level of autocorrelation (1/*f* exponent) in neural time series is a meaningful and functionally relevant physiological variable that can be accounted for in models of decision-making by allowing the spectral properties of the stochastic input to vary.

While the use of simulated 1/f noise as input to the accumulator yields a very good fit to the data (wait-time distribution and RP) there might be other ways to accomplish this. I was able to achieve a moderately good fit to the data using low-pass filtered white noise, although the best fit was still significantly poorer than that achieved using simulated 1/*f* noise. Also, the (log-log) slope of the resulting spectrum was far from that observed in the empirical data (∼3.0 for low-pass filtered noise vs ∼1.4 for simulated 1/*f* noise). So, while it might be possible to fit the wait-time distribution and/or RP using low-pass filtered white noise as input, the spectrum of the resulting input time series is a very poor match to that observed empirically. Thus, one key advantage of using simulated 1/*f* noise versus low-pass filtered white noise is that it can account not only for the behavior and event-related potential, but also for the spectral properties of the EEG data, offering a more complete representation of the data. Still, no claim is made here as to whether or not true power-law noise is required. What is required, at a minimum, is that the stochastic input time series are temporally autocorrelated and that the degree of autocorrelation can be varied parametrically. The technique used here to generate simulated 1/*f* noise (see Materials and Methods) offers a means of doing that.

Accumulator models are commonly viewed as operating at a higher level of abstraction than neural models. Nevertheless, aspects of such models are commonly mapped onto neural phenomena (Gold and Shadlen, 2007). The output of the accumulator (*x*, the decision variable) is commonly taken to represent the firing rate of neurons involved in decision-making, and the constant input (*I*) is taken to represent the sensory evidence in the form of firing rates of sensory perceptual neurons that synapse, directly or indirectly, onto the decision neurons. The noise term on the other hand is not typically considered to map directly onto a specific neural phenomenon, but rather accounts for variability within and across trials. Here, I offer a more explicit treatment of what the noise term reflects: it reflects stochastic variability in neural activity originating from elsewhere in the brain, and as such perhaps should be modeled as having the same power spectrum as real neural noise. This spectrum is known to be pink rather than white.

One might argue that, although the use of autocorrelated noise in the model is more biologically realistic, it may add relatively little in terms of accounting for and helping to explain the data. As mentioned previously, standard accumulator models, with Gaussian white input noise, have been widely used in the study of perceptual decision-making and reaction time paradigms and have proven very effective in accounting for neural and behavioral data. While this may be true, the fact that the predictions made here emerge from the model only when the input noise is autocorrelated lends credence to the argument that the spectral properties of the noise used in such models does matter. Further research will be needed to bear this out, but intuitively it seems that it will likely matter most when the imperative (the drift term) is weak relative to the noise, i.e., in the context of decision-making under uncertainty.

## Synthesis

Reviewing Editor: Gustavo Deco, Universitat Pompeu Fabra Decisions are customarily a result of the Reviewing Editor and the peer reviewers coming together and discussing their recommendations until a consensus is reached. When revisions are invited, a fact-based synthesis statement explaining their decision and outlining what is needed to prepare a revision will be listed below. The following reviewer(s) agreed to reveal their identity: Uri Maoz, Alberto Romagnoni

This manuscript is a reanalysis of previous data by Schurger and colleagues. It strives to make two points regarding the fit of drift-diffusion models (DDMs) to decision-making processes. The first relates to using pink rather than white noise as the stochastic parameter. The second, following the first, is the idea of the readiness potential (RP) as input (i.e., the noise parameter) in the model rather than as the output of the model. Both ideas are interesting and worth pursuing. And the author's analysis and results are overall convincing and, in my opinion, well worthy of publication at eNeuro.

However, before accepting this paper, I would recommend that the author add, contend with, or emphasize the following issues.

Major comments

• The manuscript is not as easy to read as it could be. The author seems to be making two points. The first is that pink noise should be used in the drift-diffusion model instead of white noise or smoothed white noise. The second is that RP-as-input in the model (assuming pink noise) is preferable to RP-as-output. While this distinction is clearer in the discussion, it is less clear in the Introduction and Results sections. The author does not make it clear enough where they refer to the first point and where to the second point. It would also be better to convince the reader more thoroughly that the first point holds and then move on to the second point. Also, it would be good to have a clearer picture of the road ahead at the end of the introduction and then another summary of where we are at the beginning of the discussion. I have read the paper more than once by now, and I am still not completely clear on the exact path that its argumentation takes.

• Following the above, a major claim of this paper is that pink noise is more appropriate for DDMs of decision-making than white noise. The author therefore needs to do more to convince the reader that this is the case. The author should either compare the outputs of the two DDMs and show that one is a better fit to actual data than the other, or they should otherwise clarify the advantages of using pink-noise DDMs. One idea for the latter might be to compare RP-as-input and RP-as-output.

• A key premise of the paper relates to the importance of a more-biological simulation of noise (i.e., pink noise) in drift-diffusion models. While this is debated, it seems that DDMs are more often viewed as higher-level mathematical abstractions rather than a direct reflection of the underlying neural hardware, which are modeled using circuit models (e.g., Xiao-Jing Wang's work at NYU; though Schurger et al. (2012) take a more literal view of the model as a representation of the RP). The author might want to discuss this relation and explain why pink noise is appropriate for this higher-level abstraction.

• The difference between the input and output modeling seems to be a key point in the paper. The author should do more to explain this difference. It takes a bit or reading of the paper to even understand that the input to the model refers to its stochastic (noise) parameter. This could be made clearer already in the abstract, by modifying “...might reflect the input to the accumulator (i.e., its stochastic noise component) rather than the output from the accumulator.”

• The author provides a simple figure that demonstrates the stochastic input and accumulator output (Fig. 2). It would be helpful to show more examples-say, with beta = 0, 1, 2, and 3. This could be done as panels B, C, D, & F of Fig. 2, each consisting of 2 axes, one above the other, and depicting a typical example of the input and the output for that value of beta.

• The pink noise is compared to white noise and to smoothed white noise. A time-domain and frequency-domain profile of the first is also depicted in Fig. 1. But the second (smoothed white noise) is only discussed in the text. It would be good to have its time- and frequency-domain profiles depicted too, perhaps in another panel of Fig. 1.

• An alternative explanation of the negative correlation between the wait time and W time might be that when people wait longer they are paying less attention to the task. So, they attribute an earlier decision-onset time, because it might have taken them longer to move after deciding to move, because they were distracted. The author might want to contend with this interpretation.

• Also, regarding W time, there is a lot of controversy about the meaning of W time and whether it even measures anything relating to the onset of intention. It would be good to mention this in the manuscript. One recent review that could potentially be useful here could be Maoz et al. “On reporting the onset of the intention to move” from 2014.

Minor comments

• The author sometimes uses ‘I’ and sometimes ‘we’. This should be consistent.

• In some places the author writes that 1<beta<3 and in other places, including Fig. 1, 0<beta<2. It seems that the former is what the author has in mind, given the methods. So, the range should be made clearer and also consistent across the manuscript. In that case, in Fig. 1, panels should be added for beta=2.5 and beta=3.It would also be good to edit the abstract to “...is temporally autocorrelated (1<beta<3), allowing...”. This would clarify the range of values of beta from the start.

• The estimation of beta exponent in the 1/f noise (in Methods) is done using linear regression in log-log coordinates. However, note that such a linear regression minimizes the overall vertical distance from the line to the samples in log space. So, it is inaccurate. These values can nevertheless be used as starting values fora direct, non-linear regression of the exponent. These approximations as starting values typically make the regression converge faster and to a good local minimum (often the global one).

• The threshold for the RP-as-input simulations is rather low (0.1256). But the right panel pf Fig. 3 has an RP reaching from 4 to <1. On the next page, it is written: “Also shown is the average time course... fit to the average readiness potential.” Is this fitting the method by which the low threshold accumulation is resized to reach a height of >3 volts? If so, this should be made clearer in the text.

• When discussing the fit of the smoothed white noise, was the same exhaustive parameter search method used as for the 1/f noise? If so, this should be mentioned. If not, the same exhaustive method should be used.

• In Fig. 5A, are the signals vertically separated for visualization purposes given the arbitrary units? If not it is not clear why the black and gray curves do not reach the same peak height. If they are, it should be clearly explained in the text. Also, in Fig. 5C&D, the short-wait input is higher than the long-wait one. It would be better to raise the gray line a bit more. The way it is now, it might seem to be the same height and differentiated slightly for visualization purposes only. It should also be clearly explained in the legend and not just the main text.

• In Fig. 6, it would be better to show the mean {plus minus} s.e.m. (or better yet, 95% confidence intervals-CI) instead of just the means and significant differences. While the latter is often done in EEG research, this does not make it any less of a bad idea statistically. Beyond the usual issues with this method, here we should also then see how the short wait CI increases for more negative time, because less trials are included.

• Fig. 8: Why not show r, p, and b for the model fit too?

• Fig. 9: What were the parameters of the binomial test that suggested that the probability of 5 out of 14 subjects would be significant at p<0.05 is 0.00003? It is not clear. However, that less than half the subjects exhibiting significant correlations does not seem that impressive. Perhaps an alternative binomial test is for the likelihood that 13 of the 14 r's would be negative, assuming a chance level of 50% for positive or negative. The p values for that is very low (p=0.0009).

• A small language point: At the very bottom of the page where Fig. 9 resides, instead of saying that it is well known that W is affected by information from before and after movement onset, it might be better to say that there is strong evidence for that.

• The author might want to discuss Casper & Cleeremans (2015). Their results on impulsivity, W time, and RP might be relevant to the results in this paper.

Review 2:

Just a very minor remark: I suggest to be more consistent throughout the paper in the range accepted for the parameter beta (sometimes between 1 and 2, sometimes between 1 and 3).

## Author Response

9 October 2017

Re.: manuscript eN-NWR-0302-17

Dear Dr. Deco and two anonymous reviewers:

Thank you for taking the time to review my submission and for providing a detailed and constructive critique. I am very glad that you consider the manuscript to be potentially suitable for publication in eNeuro. I have carefully considered each of the issues that were raised and have revised the manuscript accordingly. Below you will find a point-by-point response to the issues raised. Please note that newly added text in the manuscript will appear in red font, as will my responses below. I hope that, in its revised form, you will now find the manuscript suitable for publication in eNeuro.

Kind regards

Major issues:

• The manuscript is not as easy to read as it could be. The author seems to be making two points. The first is that pink noise should be used in the drift-diffusion model instead of white noise or smoothed white noise. The second is that RP-as-input in the model (assuming pink noise) is preferable to RP-as-output. While this distinction is clearer in the discussion, it is less clear in the Introduction and Results sections. The author does not make it clear enough where they refer to the first point and where to the second point. It would also be better to convince the reader more thoroughly that the first point holds and then move on to the second point. Also, it would be good to have a clearer picture of the road ahead at the end of the introduction and then another summary of where we are at the beginning of the discussion. I have read the paper more than once by now, and I am still not completely clear on the exact path that its argumentation takes.*

Thank you for your suggestions. I have tried to improve the readability of the manuscript by making the distinction more clear between the two main points. I have re-written and rearranged parts of the introduction and results sections taking into account your suggestions. I hope that the manuscript is now easier to read and understand.

• Following the above, a major claim of this paper is that pink noise is more appropriate for DDMs of decision-making than white noise. The author therefore needs to do more to convince the reader that this is the case. The author should either compare the outputs of the two DDMs and show that one is a better fit to actual data than the other, or they should otherwise clarify the advantages of using pink-noise DDMs. One idea for the latter might be to compare RP-as-input and RP-as-output.*

To be more precise, the claim is that allowing beta to vary (as it does in real neural systems) is a good idea for DDMs of decision making. The resulting value for beta that is suggested by the model could in principle be near zero (~ white noise) or it could be roughly between 1 and 2 (pink noise). The point is that we have added a new parameter, a new dimension, to the model allowing it to account for properties of the data that were previously ignored, potentially leading to new predictions. One major advantage of allowing beta to vary is that it causes the input to behave in a way that is amenable to modeling. I.e. the comparison between the RP-as-input and RP-as-output variants of the model only becomes interesting when beta is allowed to vary. Another advantage is that with variable beta we can account more fully for the neural data, by fitting its spectral properties. This led to new testable predictions. I have added some text to the discussion section clarifying the advantages of allowing the spectrum of the noise to vary in DDMs.

• A key premise of the paper relates to the importance of a more-biological simulation of noise (i.e., pink noise) in drift-diffusion models. While this is debated, it seems that DDMs are more often viewed as higher- level mathematical abstractions rather than a direct reflection of the underlying neural hardware, which are modeled using circuit models (e.g., Xiao-Jing Wang's work at NYU; though Schurger et al. (2012) take a more literal view of the model as a representation of the RP). The author might want to discuss this relation and explain why pink noise is appropriate for this higher-level abstraction.*

It is true that DDMs are commonly viewed as operating at a higher level of abstraction than neural models. Nevertheless, aspects of the DDM are very commonly mapped directly onto neural phenomena (e.g. the work of Mike Shadlen and others). The output of the DDM (the decision variable) is commonly taken to represent the firing rate of neurons involved in decision making, and the drift term is seen as representing the sensory evidence and reflects the firing rates of sensory perceptual neurons that synapse, directly or indirectly, onto the decision neurons. But the noise term is not typically considered to map directly onto a specific neural phenomenon, but rather represents the more abstract notion of "noise in the system". I am hoping to change that view by being more explicit about what the noise term reflects: it reflects noise in neural firing rates coming from elsewhere in the brain, and as such it should be modeled as having the same power spectrum as real neural noise. This spectrum is known to be pink rather than white.

I have added the above to the discussion section.

• The difference between the input and output modeling seems to be a key point in the paper. The author should do more to explain this difference. It takes a bit or reading of the paper to even understand that the input to the model refers to its stochastic (noise) parameter. This could be made clearer already in the abstract, by modifying “...might reflect the input to the accumulator (i.e., its stochastic noise component) rather than the output from the accumulator.”*

I have made the suggested change to the abstract and in revising the intro, results, and discussion I hope that I have now made this more clear.

• The author provides a simple figure that demonstrates the stochastic input and accumulator output (Fig. 2). It would be helpful to show more examples-say, with beta = 0, 1, 2, and 3. This could be done as panels B, C, D, & F of Fig. 2, each consisting of 2 axes, one above the other, and depicting a typical example of the input and the output for that value of beta.*

Thank you for this suggestion. I have made the requested modifications to figure 2, adding five additional panels, except that I used beta = 0, 0.5, 1.0, 1.5, and 2.0 and I also added a panel for the lowpass filtered input. I chose those values for beta mainly because the average input and output both get more or less linear for beta > 2.0 (when time locked to threshold crossing), so the interesting part of the range is roughly between 0 and 2. Also this makes figure 2 consistent with figure 1, which now shows the time series and spectra for these same values of beta.

• The pink noise is compared to white noise and to smoothed white noise. A time-domain and frequency- domain profile of the first is also depicted in Fig. 1. But the second (smoothed white noise) is only discussed in the text. It would be good to have its time- and frequency-domain profiles depicted too, perhaps in another panel of Fig. 1.*

I have re-done figure 1 so that it now has six panels: one each for five different values of beta (0, 0.5, 1.0, 1.5, 2.0) plus one for the lowpass filtered input. So now figures 1 and 2 are nicely aligned: fig. 1 shows a sample time series and power spectrum for five values of beta plus lowpass filtered, and fig. 2 shows the averaged input and averaged output of the accumulator for the same five values of beta plus lowpass filtered.

• An alternative explanation of the negative correlation between the wait time and W time might be that when people wait longer they are paying less attention to the task. So, they attribute an earlier decision-onset time, because it might have taken them longer to move after deciding to move, because they were distracted. The author might want to contend with this interpretation.*

I am not sure that I fully understand why paying less attention to the task should imply an earlier rather than later decision onset time. E.g. one could just as well argue that paying less attention might lead subjects to fail to notice their decision and so revert to reporting the time of movement onset rather than W time. Also, if I have understood correctly, this interpretation rests on the assumption that attention becomes less focused with time (on the order of seconds). While this seems intuitive, it is not necessarily true in every context. One indirect way to check this is to repeat the analysis for the first half and second half of the experiment. If attention is waning over time, and attention explains the effect, then the mean W time might become earlier in the second half of the experiment compared to the first half. I tested this and there is no sign of a difference between the two (p > 0.8 for a matched-samples t-test or a signed rank test), although the main result remains significant (p < 0.01) in both halves of the experiment. Of course it could still be the case that attention wanes locally over short time scales (i.e. during a trial) even if not over longer time scales (over the course of the experiment). But this analysis at least helps to bolster confidence in our interpretation. If attention does covary with waiting time on the time scale of a trial then it will require a different experimental design in order to control for it. One final point is that this novel effect was predicted by the model. Even if there are other potential explanations for the effect, this does not diminish the fact that the model guided us to look at waiting time.

• Also, regarding W time, there is a lot of controversy about the meaning of W time and whether it even measures anything relating to the onset of intention. It would be good to mention this in the manuscript. One recent review that could potentially be useful here could be Maoz et al. “On reporting the onset of the intention to move” from 2014.

Thank you for directing me to the Maoz et al. article, which I found to be an excellent synthesis of the literature and debate about W time. While there has justifiably been a lot of controversy surrounding W time, and I share your skepticism, there are a couple of crucial data points that make it difficult to argue that W time does not measure anything meaningfully related to intentions. For example, Sirigu et al. (2004) found that W time in parietal patients was significantly closer to movement onset than in normals or cerebellar patients, and the effect was specific to W time: there was no effect on M time. More recently Douglas et al. (2015) were able to selectively influence W time judgements using tDCS applied to the angular gyrus or to M1, but not to the SMA. tDCS did not change M time judgements or judgements of the time of an auditory stimulus. When analyzing the LRP these authors found effects from both before and after movement onset that covaried with W time judgements. The specificity of these effects on W time judgements make it difficult to believe that W time judgements are arbitrary or meaningless. I now mention the controversy surrounding W time in the Discussion section.

Minor points:

• The author sometimes uses ‘I’ and sometimes ‘we’. This should be consistent.*

Thank you for pointing that out - this was an oversight on my part. I now use 'I' throughout, except where I specifically intended to use the word 'we' to refer to "the reader and I" or to "people in general".

• In some places the author writes that 1<beta<3 and in other places, including Fig. 1, 0<beta<2. It seems that the former is what the author has in mind, given the methods. So, the range should be made clearer and also consistent across the manuscript. In that case, in Fig. 1, panels should be added for beta=2.5 and beta=3.It would also be good to edit the abstract to “...is temporally autocorrelated (1<beta<3), allowing...”. This would clarify the range of values of beta from the start.*

I see how this might be confusing - thanks for pointing it out. I have changed all but one instances to "1 < beta < 3" for consistency. In one instance (first paragraph of the discussion) I use "0 <= beta <= 3" because here I am referring to the possible values that beta can take on in this context, whereas in other instances I was referring to the values that beta typically takes on in this context. As for figure 1, I chose to focus on the range from 0 <= beta <= 2 because (1) I wanted to start with white noise (beta=0) and (2) I wanted merely to illustrate qualitatively how the signal changes as beta changes. Adding panels for 2.5 and 3.0 would make a large dense figure even larger (there are already 12 panels). This also makes it so that figures 1 and 2 both show panels for the same set of beta values. I have added the following text to the figure caption in order to clarify: "Time series are shown for values of beta between 0 and 2 in order to illustrate the way that time series change qualitatively as beta changes."

• The estimation of beta exponent in the 1/f noise (in Methods) is done using linear regression in log-log coordinates. However, note that such a linear regression minimizes the overall vertical distance from the line to the samples in log space. So, it is inaccurate. These values can nevertheless be used as starting values fora direct, non-linear regression of the exponent. These approximations as starting values typically make the regression converge faster and to a good local minimum (often the global one).*

The "correct" way to estimate beta depends on whether you think error should be minimized in the log-log space or the linear space. Given that the log-log space description is the most parsimonious (linear), one could argue that minimizing error in the log-log space makes the most sense. This seems to be the consensus in the field because all but one of the commonly-used estimators of the 1/f exponent (fft, Welch, DFA, wavelet) includes some form of linear fitting in a log-log space. The Whittle method does not, but that method seems to be undefined for beta > 1, so is not suitable for our data where beta varies from ~0.5 to ~2.0.

Also, I should point out that the estimation of beta in the empirical data is not crucial for either of the main findings. It is used only to show that the empirically observed value for beta agrees with that suggested by the model, and since they are both estimated in the same way then at least the comparisons are fair. So, in summary, I have tried to argue in favor of using linear regression in a log-log space.

• The threshold for the RP-as-input simulations is rather low (0.1256). But the right panel pf Fig. 3 has an RP reaching from 4 to <1. On the next page, it is written: “Also shown is the average time course... fit to the average readiness potential.” Is this fitting the method by which the low threshold accumulation is resized to reach a height of >3 volts? If so, this should be made clearer in the text.*

Sorry that this was unclear. Yes, the fitting procedure includes a scaling factor whereby the amplitude of the simulated RP is scaled to that of the empirical RP. I now mention this explicitly in the figure caption and also in the main text.

• When discussing the fit of the smoothed white noise, was the same exhaustive parameter search method used as for the 1/f noise? If so, this should be mentioned. If not, the same exhaustive method should be used.

Thank you for pointing this out. Yes, it was the same exhaustive search method. I now explicitly state this in the text.

• In Fig. 5A, are the signals vertically separated for visualization purposes given the arbitrary units? If not it is not clear why the black and gray curves do not reach the same peak height. If they are, it should be clearly explained in the text. Also, in Fig. 5C&D, the short-wait input is higher than the long-wait one. It would be better to raise the gray line a bit more. The way it is now, it might seem to be the same height and differentiated slightly for visualization purposes only. It should also be clearly explained in the legend and not just the main text.

In fig. 5A the signals are not vertically separated for visualization purposes. This is the way the data came out of the simulation. The two curves in 5A do not necessarily have to reach the same height at t0 because these are the average inputs to the accumulator. I.e. their integrals have to reach the same height at t0 (which is the case - see fig. 5B). Note that, because the epochs are time locked to threshold crossings in the output, only the outputs are guaranteed to reach the same height at t0. The vertical separation of the input lines in 5 C & D is by design. The short-wait input line is twice as high as the long-wait line so that the slope of the short-wait output line will be 2x that of the long-wait output line. This makes everything fit nicely on the axis. If the slope of the output line is too steep, then in panel D there will not be much of the gray lines left on the axis. I have added text to the figure caption clearly explaining the reason for the separation of the different curves in this figure.

• In Fig. 6, it would be better to show the mean {plus minus} s.e.m. (or better yet, 95% confidence intervals- CI) instead of just the means and significant differences. While the latter is often done in EEG research, this does not make it any less of a bad idea statistically. Beyond the usual issues with this method, here we should also then see how the short wait CI increases for more negative time, because less trials are included.

Fig. 6 now shows the mean +/- s.e.m. as well as the significant differences.

• Fig. 8: Why not show r, p, and b for the model fit too?*

That was an oversight on my part. Thank you for pointing it out. I now show r, p, and b for both the model fit and the empirical data.

• Fig. 9: What were the parameters of the binomial test that suggested that the probability of 5 out of 14 subjects would be significant at p<0.05 is 0.00003? It is not clear. However, that less than half the subjects exhibiting significant correlations does not seem that impressive. Perhaps an alternative binomial test is for the likelihood that 13 of the 14 r's would be negative, assuming a chance level of 50% for positive or negative. The p values for that is very low (p=0.0009).*

For the binomial test, I specified 5 as the number of successes, 14 as the total number of tries, and 0.05 as the probability of success. However, your formulation of reporting the probability that 13 out of 14 r's would be negative seems more intuitive. So in the manuscript I now report that probability.

• A small language point: At the very bottom of the page where Fig. 9 resides, instead of saying that it is well known that W is affected by information from before and after movement onset, it might be better to say that there is strong evidence for that.*

I made this change to the text.

• The author might want to discuss Casper & Cleeremans (2015). Their results on impulsivity, W time, and RP might be relevant to the results in this paper.

I have added a brief discussion of the Caspar and Cleereman's paper in the discussion section.

Review 2:

Just a very minor remark: I suggest to be more consistent throughout the paper in the range accepted for the parameter beta (sometimes between 1 and 2, sometimes between 1 and 3).

I have made sure to use the same range for beta (1 - 3) throughout the paper.
